# Tissue Distribution and Pharmacokinetic Characteristics of Aztreonam Based on Multi-Species PBPK Model

**DOI:** 10.3390/pharmaceutics17060748

**Published:** 2025-06-06

**Authors:** Xiao Ye, Xiaolong Sun, Jianing Zhang, Min Yu, Nie Wen, Xingchao Geng, Ying Liu

**Affiliations:** Institute for Safety Evaluation, National Institutes for Food and Drug Control, Beijing Key Laboratory for Safety Evaluation of Drugs, Beijing 100176, China; yexiao@nifdc.org.cn (X.Y.); sunxiaolongyeah@163.com (X.S.); zhangjianing@nifdc.org.cn (J.Z.); yumin@nifdc.org.cn (M.Y.); wennie@nifdc.org.cn (N.W.)

**Keywords:** physiologically based pharmacokinetic model, aztreonam, concentration-time profile, multi-species extrapolation

## Abstract

**Background/Objectives**: As a monocyclic β-lactam antibiotic, aztreonam has regained attention recently because combining it with β-lactamase inhibitors helps fight drug-resistant bacteria. This study aimed to systematically characterize the plasma and tissue concentration-time profiles of aztreonam in rats, mice, dogs, monkeys, and humans by developing a multi-species, physiologically based pharmacokinetic (PBPK) model. **Methods**: A rat PBPK model was optimized and validated using plasma concentration-time curves determined by liquid chromatography–tandem mass spectrometry (LC-MS/MS) following intravenous administration, with reliability confirmed through another dose experiment. The rat model characteristics, modeling experience, ADMET Predictor (11.0) software prediction results, and allometric scaling were used to extrapolate to mouse, human, dog, and monkey models. The tissue-to-plasma partition coefficients (*K_p_* values) were predicted using GastroPlus (9.0) software, and the sensitivity analyses of key parameters were evaluated. Finally, the cross-species validation was performed using the average fold error (AFE) and absolute relative error (ARE). **Results**: The cross-species validation showed that the model predictions were highly consistent with the experimental data (AFE < 2, ARE < 30%), but the deviation of the volume of distribution (*V_ss_*) in dogs and monkeys suggested the need to supplement the species-specific parameters to optimize the prediction accuracy. The *K_p_* values revealed a high distribution of aztreonam in the kidneys (*K_p_* = 2.0–3.0), which was consistent with its clearance mechanism dominated by renal excretion. **Conclusions**: The PBPK model developed in this study can be used to predict aztreonam pharmacokinetics across species, elucidating its renal-targeted distribution and providing key theoretical support for the clinical dose optimization of aztreonam, the assessment of target tissue exposure in drug-resistant bacterial infections, and the development of combination therapy strategies.

## 1. Introduction

The advent of antibiotics has revolutionized the treatment of bacterial infections and significantly reduced the mortality rates associated with these pathogens [1]. However, with the widespread and improper application of antibiotics, the problem of bacterial resistance has become increasingly prominent, which not only reduces the clinical use effect but also poses a major threat to global public health [2,3]. Among the numerous resistance mechanisms, the enzymatic degradation mediated by β-lactamases, particularly extended-spectrum β-lactamases (ESBLs) and metallo-β-lactamases (MBLs), represents one of the core reasons for clinical treatment failure. These enzymes effectively inactivate β-lactam antibiotics, including penicillins and cephalosporins, through the hydrolysis of their essential β-lactam ring structure [4,5]. In the face of this predicament, the development of drugs with both broad-spectrum antibacterial activity and the ability to overcome resistance is an urgent need.

Aztreonam is a rather special member of the β-lactam antibiotics. It was developed by SmithKline Beecham Corporation of the United States and was approved for the market in 1984. To date, it remains the only monocyclic β-lactam antibiotic in clinical use [6,7]. Initially, this drug was widely applied in the treatment of various infections caused by aerobic Gram-negative bacteria, such as urinary tract infections, respiratory tract infections, and abdominal infections. Unlike most β-lactam antibiotics (e.g., penicillins and cephalosporins), aztreonam exhibits stability against hydrolysis mediated by metallo-β-lactamases (MBLs, Class B β-lactamases) [8], thereby demonstrating unique advantages in treating infections caused by MBL-producing multidrug-resistant Gram-negative bacteria. In recent years, studies on the combination of aztreonam with new β-lactamase inhibitors, particularly avibactam, have attracted attention [9]. Research shows that aztreonam combined with various new β-lactamase inhibitors has strong antibacterial activity against Enterobacteriaceae producing metallo-β-lactamases, among which the combination of aztreonam and avibactam has demonstrated a particularly significant antibacterial effect. Currently, the European Medicines Agency (EMA) has recommended Emblaveo^®^ (aztreonam/avibactam) for marketing in the European Union for the treatment of Gram-negative complications caused by multi-drug-resistant infections, while both the European Society of Clinical Microbiology and Infectious Diseases (ESCMID) and the American Society for Infectious Diseases (IDSA) have listed it as a key strategy against MBL-mediated resistance [10]. Although aztreonam is an antibiotic with a long history, through the its combined application with new β-lactamase inhibitors, it still demonstrates significant value in combating multi-drug-resistant bacterial infections [11].

From the perspective of pharmacokinetic characteristics, aztreonam exhibits poor oral bioavailability, but it can be rapidly absorbed after an intramuscular injection, with a bioavailability close to 100%. It is mainly excreted unchanged through urine, and it has a relatively short plasma half-life, ranging from 1.3 to 2.2 h in adults with normal renal function [6,8]. However, early studies were limited by their research techniques and ethical considerations, resulting in very limited data on the distribution of aztreonam in human tissues. Furthermore, the preclinical pharmacokinetic data of aztreonam were conducted a long time ago [12,13,14] and lack direct translational relevance to human physiology. The physiologically based pharmacokinetic (PBPK) model, which integrates anatomical, physiological, and biochemical parameters, provides a new opportunity for the in-depth exploration of aztreonam’s pharmacokinetic behavior. The PBPK model can precisely simulate the complex transport process of drugs in the body and effectively overcome the limitations of traditional research methods, predicting the concentration-time profiles characteristics of drugs in different tissues [15,16].

Thus, this study establishes a novel PBPK model to systematically characterize the plasma and tissue pharmacokinetics of aztreonam across species. The results serve as a paradigm for establishing a scientific framework to guide rational clinical applications and accelerate the development of aztreonam derivatives and related antimicrobial agents.

## 2. Materials and Methods

### 2.1. Chemicals and Reagents

The aztreonam standard (purity: 96.9%) was obtained from the National Institutes for Food and Drug Control (NIFDC, Beijing, China). The internal standard (IS, aztreonam structural analog, purity: 80.0%) was custom-synthesized in our laboratory. Acetonitrile, methanol, isopropanol, and formic acid (LC-MS grade) were purchased from Fisher Scientific (Hampton, NH, USA). Ultrapure water was generated by a Milli-Q Advantage A10 system (Millipore, Burlington, MA, USA).

### 2.2. Experimental Animals and Sample Collection

#### 2.2.1. Rat Experiment

Twelve Sprague–Dawley (SD) rats (8–9 weeks old; equal numbers of males and females; female body weight: 200–300 g, male body weight: 300–400 g) were provided by Vital River Laboratory Animal Technology Co., Ltd. (Beijing, China; License No. SCXK [Jing] 2016-0011). The animals were housed in an SPF-grade barrier environment with controlled conditions: temperature 20–26 °C (daily fluctuation ≤ 3 °C), relative humidity 40–70%, ventilation rate ≥ 15 air changes per hour, and a 12 h light/dark cycle. All procedures were approved by the Institutional Animal Care and Use Committee (IACUC) of the National Drug Safety Evaluation Center (Approval No. IACUC-2023-078).

Aztreonam (50 mg/kg) was administered as a single intravenous bolus to rats. The formulation was prepared by dissolving the aztreonam active pharmaceutical ingredient (API) in sterile water for injection at 10 mg/mL, followed by filtration through a 0.2 μm membrane, and storage protected from light at 2–8 °C for up to 24 h before use. Blood samples were collected before dosing and at 15 min, 30 min, 1 h, 2 h, 4 h, 7 h, 10 h, and 24 h post-dose. At each time point, blood was drawn from three rats into EDTA-2K-coated tubes and centrifuged at 4000× *g* for 10 min to isolate plasma. The plasma samples were stored at −70 °C until the analysis.

#### 2.2.2. Data of Mice, Dogs, Monkeys, and Humans

The pharmacokinetic data of aztreonam in mice, dogs, and monkeys were obtained by comprehensively searching databases on the Web of Science and PubMed. Using “aztreonam”, “mouse/dog/monkey”, and “pharmacokinetics” as keywords, relevant research literature was screened to extract the plasma and tissue concentration-time data. The human pharmacokinetic data were collected from published clinical trial literature (Table 1).

### 2.3. Analytical Method Development

#### 2.3.1. LC-MS/MS Analysis

The concentration of aztreonam in rat plasma was quantified using a Waters Xevo TQ-XS liquid chromatography–tandem mass spectrometry (LC-MS/MS) system.

The separation was performed on a Waters XSelect HSS T3 column (2.1 mm × 50 mm, 2.5 µm; Waters, Milford, MA, USA) equipped with a 0.2 µm inline filter. The mobile phase consisted of 0.01% aqueous formic acid (A) and acetonitrile (B) delivered at a flow rate of 0.4 mL/min with the following gradient program: 0–1 min, 5% B; 1–1.5 min, 5–60% B; 1.5–2.5 min, 60–95% B; 2.5–4 min, 95% B; 4.01–5 min, 5% B. The column temperature was maintained at 40 °C, and the injection volume was 2 μL with an autosampler temperature of 10 °C.

Electrospray ionization in the negative mode (ESI¯) was employed with the following parameters: ion source temperature 150 °C, capillary voltage 0.5 kV, desolvation gas temperature 550 °C, desolvation gas flow 1000 L/h, cone gas flow 150 L/h, nebulizer pressure 7.0 Bar, and collision gas (argon) flow 0.15 mL/min. The cone voltage for both aztreonam and the internal standard (IS) was set to 20 V, with collision energies of 18 eV (aztreonam) and 15 eV (IS). The quantification was performed in the multiple reaction monitoring (MRM) mode using the transitions *m*/*z* 434.1 → 95.9 for aztreonam and *m*/*z* 462.0 → 235.9 for the IS.

#### 2.3.2. Sample Preparation

Aliquots of plasma samples were diluted appropriately with blank SD rat plasma. For analysis, 50 μL of diluted plasma or undiluted plasma was transferred to a microcentrifuge tube. Next, 200 μL of methanol–acetonitrile (9:1, *v*/*v*) containing an internal standard (60 ng/mL) was added. The mixture was vortex-mixed for 2 min and centrifuged at 13,200× *g* for 15 min. A 2 μL aliquot of the supernatant was injected into the LC-MS/MS system for analysis.

#### 2.3.3. Method Validation

The bioanalytical method was validated according to the M10: Bioanalytical Method Validation and Study Sample Analysis guideline issued by the International Council for Harmonisation of Technical Requirements for Pharmaceuticals for Human Use (ICH). Detailed validation results are provided in the Supplementary Materials.

### 2.4. PBPK Model Building

#### 2.4.1. Modeling Strategy and Parameter Prediction

The chemical structure of aztreonam (molecular formula: C_13_H_17_N_5_O_8_S_2_; CAS No. 78110-38-0) was imported into ADMET Predictor^®^ 11.1.1 (Simulations Plus, Inc., Lancaster, CA, USA) to predict its key physicochemical parameters (Table 2) and metabolic characteristics. The results indicated that aztreonam is primarily excreted unchanged via renal mechanisms (S+ Mechanistic Clearance Classification = Renal) and exhibits potential biliary excretion [P-glycoprotein (P-gp) substrate probability: 99%; Breast Cancer Resistance Protein (BCRP) substrate probability: 67%]. No significant metabolism mediated by CYP450 enzymes was observed.

Based on these parameters, the drug properties were imported into the “Compound Properties” module of GastroPlus™ 9.0 (Simulations Plus, Inc., Lancaster, CA, USA) to establish the initial inputs for the pharmacokinetic model construction.

- Rat, Mouse, and Human Models

**Figure 1:**
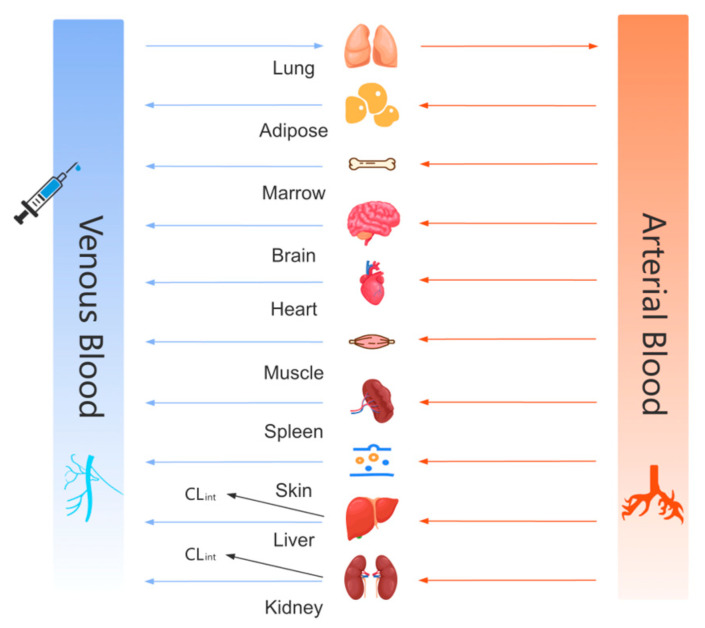
The model structure of the physiologically based pharmacokinetic (PBPK) model after the intravenous injection of aztreonam.

The physiological parameters (e.g., organ volumes and hemodynamic parameters) were retrieved from the built-in physiological database of GastroPlus™ (Figure 1). For rats, the model was calibrated using experimentally measured body weights (250 ± 20 g), with physiological parameters automatically scaled to the corresponding body weight (see Table 3). The renal filtration clearance *CL_renal_* was defined as *f_up_* (plasma free fraction) × GFR (glomerular filtration rate), and the biliary excretion was assigned a fraction of 10% of the hepatic clearance (Biliary Clearance Fraction).

- Dog and Monkey Models

The species-specific physiological parameters were directly retrieved from the software’s embedded database (Table 3).

- Metabolic Assumptions

Based on the ADMET Predictor^®^ (11.0) results, the metabolic mechanisms in dogs and monkeys were assumed to align with those in rats/humans. Aztreonam was hypothesized to undergo a predominantly unchanged renal excretion (*CL_renal_* = GFR × *f_up_*), with biliary excretion (mediated by P-glycoprotein [P-gp] and Breast Cancer Resistance Protein [BCRP]) proportionally set as described above.

#### 2.4.2. Model Optimization and Cross-Species Validation

The rat pharmacokinetic model was refined based on experimentally measured plasma concentration-time data. A non-compartmental analysis (NCA) was employed to calculate the pharmacokinetic parameters (e.g., total clearance *CL*, steady-state volume of distribution *V_ss_*) for optimizing the PBPK model. First, the hepatic clearance (*CL_hep_*) and *CL_renal_* were adjusted by weighting coefficients to ensure their summation matched the *CL_tot_* derived from the NCA, thereby maintaining the physiological plausibility of systemic clearance. Second, a sensitivity analysis was conducted to identify the critical parameters (e.g., the oil–water partition coefficient [*log P*], *f_up_*) influencing tissue-to-plasma partition coefficients (*K_p_*). These parameters were selectively optimized to align the PBPK-predicted *V_ss_* with the NCA results while retaining the initial literature/experimental values for non-sensitive parameters to preserve the biological relevance. Finally, the refined model was validated against literature-reported in vivo pharmacokinetic data at 20 mg/kg [12]. The predictive performance was evaluated by comparing simulated plasma concentration-time profiles with experimental data, systematically assessing the model robustness and dose dependency.

The tissue distribution was characterized by the *K_p_*, defined as the ratio of tissue-to-plasma drug concentrations at steady state. A whole-body PBPK model was systematically developed using literature-reported rat tissue distribution data (concentration-time profiles for kidney, liver, lung, and spleen) combined with the Rodgers and Rowland single-parameter method in GastroPlus™ 9.0. The initial *K_p_* values were calculated using physicochemical properties (*Log P*, *pKa*, blood–plasma concentration ratio [*R_bp_*]) predicted by ADMET Predictor^®^ 11.0. The model accuracy was verified by comparing the predicted *K_p_* values with the experimental tissue concentration data. For tissues with an absolute relative error (ARE) exceeding 30% (e.g., liver, kidney), tissue-specific parameters (e.g., *R_bp_*, *f_up_*) were adjusted under physiological constraints to prioritize the calibration of significantly deviated distribution features. The plasma pharmacokinetic parameters (the area under the plasma concentration-time curve [*AUC*], *CL*) and *V_ss_* predictions were cross-validated against the experimental data. The model optimization strictly adhered to the rat-specific physiological data (organ volumes, blood flow rates) and literature-reported tissue composition values to avoid overfitting.

For organs lacking experimental data (e.g., brain, heart, muscle), the *K_p_* values were predicted using the same algorithm, assuming physiological similarity in passive diffusion-driven tissue distribution between rats and humans. During the human extrapolation, the drug-specific physicochemical properties (*Log P*, *pKa*) were retained, while the rat physiological parameters (organ volumes, blood flow rates) were replaced with human-specific values. The systemic clearance was scaled using allometric principles. The human tissue distribution (*K_p,Human_*) [17] was calculated as follows:

(1) $$K_{p,Human}=\frac{f_{up,human}}{f_{up,rat}}\times \frac{R_{bp,rat}}{R_{bp,human}}\times K_{p,rat}$$

to account for interspecies differences in plasma protein binding.

Based on the rat PBPK model, the mouse and human models were rapidly constructed via direct extrapolation. For the mice, the physiological parameters (organ volumes, blood flow rates, plasma protein binding) were replaced with mouse-specific values. The *K_p_* values were directly inherited from rats under the assumption of interspecies similarity in tissue composition, with adjustments for plasma protein binding differences using the free drug hypothesis. The hepatic/renal clearance rates were fine-tuned using the mouse experimental data. For the human models, the distribution and clearance mechanisms of the rats were retained, while the human physiological parameters (e.g., liver weight, cardiac output) and plasma protein binding rates were substituted. The systemic clearance was estimated via the allometric scaling formula [18,19]:

(2) $${{CL}_{Human}=CL}_{rat}\times {\frac{{Weight}_{human}}{{Weight}_{rat}}}^{0.6\sim 0.8}$$

The final extrapolation was completed by calibrating the key parameters against the clinical plasma concentration data. This approach assumes conserved core mechanisms of drug distribution and metabolism across species, significantly reducing the modeling complexity.

Due to the inability of ADMET Predictor^®^ (11.0) to predict species-specific parameters (e.g., *R_bp_*, *f_up_*) for dogs and monkeys, human or rat-derived *f_up_* and *R_bp_* values were adopted. An allometric scaling model integrating pharmacokinetic data from mice, rats, and humans was constructed to predict the *CL* and *V_ss_* in cynomolgus monkeys. Considering the physiological peculiarities of dogs [20] (such as the high muscle mass proportion), the value of *K_p_* in the muscle tissue was increased by 30%. The dog model was calibrated using literature-reported intravenous injection data following the same refinement methodology as the rat model.

#### 2.4.3. Model Evaluation Criteria

- Goodness-of-Fit Assessment

The predictive performance of the PBPK model was evaluated by the linear regression of predicted versus observed values to calculate the coefficient of determination (*R*^2^). The prediction accuracy was quantified using the fold error (FE), average fold error (AFE), and average absolute fold error (AAFE), defined as follows:

(3) $$\mathrm{FE}=\{\frac{predicted}{observed}\}$$

(4) $$AFE=10^{\sum_{i=1}^{n}\log(\frac{\mathrm{predicted}}{\mathrm{observed}})/n}$$

(5) $$AAFE=10^{\sum_{i=1}^{n}\mathrm{abs}(\log(\frac{\mathrm{predicted}}{\mathrm{observed}}))/n}$$

where Predicted and Observed represent the predicted and measured concentrations at each time point, respectively, and *n* is the total number of valid time points. The FE quantifies the deviation of the individual predictions (Equation (1)), AFE reflects the overall bias direction (systematic underprediction if AFE < 1 or overprediction if AFE > 1; Equation (2)), and AAFE measures the absolute error magnitude (Equation (3)). A model is considered acceptable if *R*^2^ ≥ 0.8 [21,22,23]. The predictions are deemed reliable if the FE values for all time points fall within 0.3–3 and both the AFE and AAFE are < 2 [24,25].

- Model Validation

The key pharmacokinetic parameters (the area under the concentration-time curve from time zero to time t [*AUC*_0–_*_t_*], the area under the concentration-time curve from time zero to infinity [*AUC*_0–∞_], *CL*, and *V_ss_*) were evaluated using the relative prediction error (RPE) and absolute relative error (ARE):

(6) $$RPE=\frac{Predicted-Observed}{Observed}\times 100\%$$

(7) $$ARE=\left|\frac{Predicted-Observed}{Observed}\right|\times 100\%$$

The model is considered reliable if the ARE values for all parameters are less than 30% [26,27] (predictions deviate from observations by less than ± 30%). The *C_max_* was excluded as a critical parameter because intravenous dosing results in an instantaneous peak concentration at time zero, whereas experimental blood sampling unavoidably lags behind this theoretical maximum.

#### 2.4.4. Parameter Sensitivity Analysis

Through the parameter sensitivity analysis, the key parameters that affect the pharmacokinetic characteristics of aztreonam in PBPK models of different species were analyzed. In this study, based on the characteristic that aztreonam directly enters the systemic circulation through intravenous administration, five core parameters, namely *Log P*, *f_up_*, *R_bp_*, *CL_renal_*, and *CL_hep_*, were selected for the sensitivity analysis. The impact extent of each parameter variation on the core pharmacokinetic indicators, such as t *AUC*_0–*∞*_, was quantitatively evaluated. The key sensitive parameters that drive the model prediction variation were systematically identified.

## 3. Results

### 3.1. Method Validation Results

The established bioanalytical method for aztreonam in rat plasma demonstrated compliance with the ICH M10 guidelines. The method exhibited high selectivity with minimal interference from endogenous components. The calibration curve showed linearity over the range of 5–1000 ng/mL, with a lower limit of quantification (LLOQ) of 5 ng/mL and an upper limit of quantification (ULOQ) of 1000 ng/mL. The intra-day and inter-day precision (%RSD) and accuracy (%bias) were within acceptable limits. Additionally, the matrix effects, dilution integrity, stability, and injection reproducibility met the predefined criteria, confirming the method’s reliability and precision for quantifying aztreonam in rat plasma. Detailed results are provided in the Supplementary Materials.

### 3.2. Pharmacokinetic Characteristics and Model Validation in Rats

#### 3.2.1. Pharmacokinetic Results in Rats

After the methodological validation was completed, a pharmacokinetic study on the single intravenous injection of 50 mg/kg aztreonam in rats was conducted. Plasma samples collected at different time points were analyzed and detected by LC-MS/MS. After a single intravenous injection of 50 mg/kg aztreonam in rats, due to the direct entry of the drug into the blood circulation, the *C_max_* was reached immediately after administration, with a value of 265.98 ± 39.09 μg/mL. Subsequently, the concentration of aztreonam in plasma began to gradually decrease, and the rate of decrease was relatively obvious within 2 h. Afterward, the downward trend became more moderate. Through the Phoenix WinNonlin (V8.3) software, an NCA was selected for analysis. The main pharmacokinetic parameters measured in the experiment were as follows: the elimination half-life (*t*_1/2_) was 1.24 ± 1.09 h; *AUC*_0–24 *h*_ was 53.61 ± 5.35 h·μg/mL; AUC_0–*∞*_ was 53.62 ± 5.37 h·μg/mL; *CL* was 0.21 ± 0.0021 L/h/kg; and *V_ss_* was 0.064 ± 0.0025 L.

#### 3.2.2. PBPK Model in Rats

**Figure 2:**
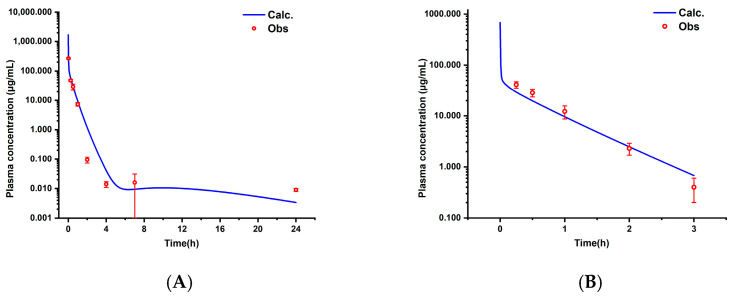
The observed and predicted concentration-time curves of aztreonam in rats (i.v.) of 50 mg/kg (**A**) and 20 mg/kg (**B**), (mean ± SD, *n* = 6).

**Figure 3:**
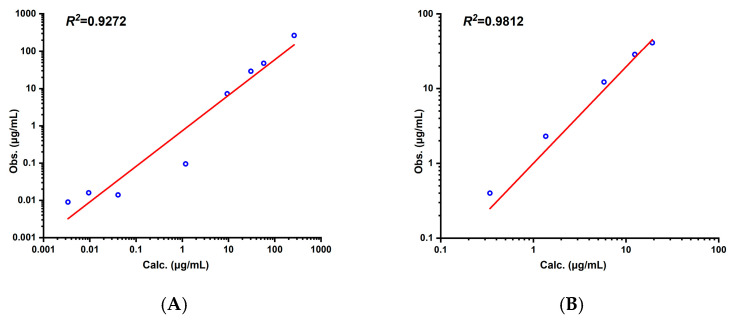
Correlation between observed and predicted values of aztreonam in rats (i.v.) of 50 mg/kg (**A**) and 20 mg/kg (**B**).

The optimized PBPK model based on the experimental data demonstrated high consistency between the predicted and observed values (*R*^2^ = 0.93, AFE = 1.02, AAFE = 1.40), as shown in Figure 2 and Figure 3. The key pharmacokinetic parameters exhibited an ARE < 30% (Table 4), with deviations of 3.4% for the *AUC*_0–*∞*_, 2.4% for the *CL*, and 6.3% for the *V_ss_*, confirming high predictive accuracy. When extrapolated to a literature-reported dose of 20 mg/kg, the model maintained strong performance (*R*^2^ = 0.98, AFE = 0.93, AAFE = 1.36), with all key pharmacokinetic parameter ARE values less than 30% (Table 4), validating its dose linearity.

**Figure 4:**
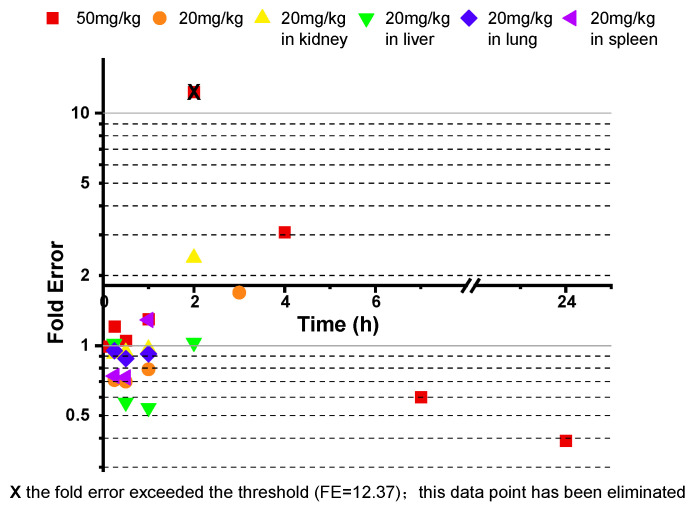
The fold error of all of the observed and predicted concentration points in the rat model.

During the PBPK model fitting for 50 mg/kg aztreonam in rats, the 2 h time point exhibited a significantly elevated fold error (FE = 12.37), exceeding the acceptable threshold (FE > 3), and it was identified as an outlier. To ensure model robustness, this data point was excluded from the final parameter optimization process. The FE results for all other time points are presented in Figure 4.

**Figure 5:**
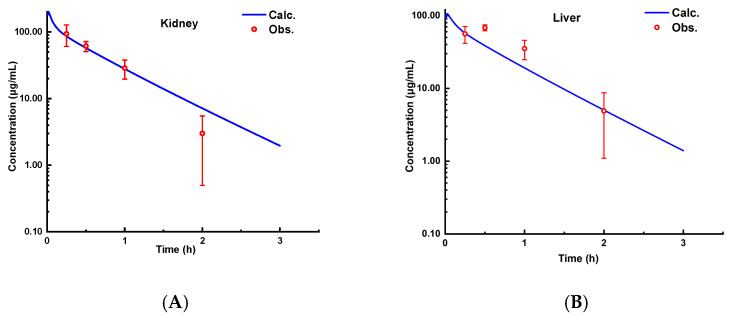

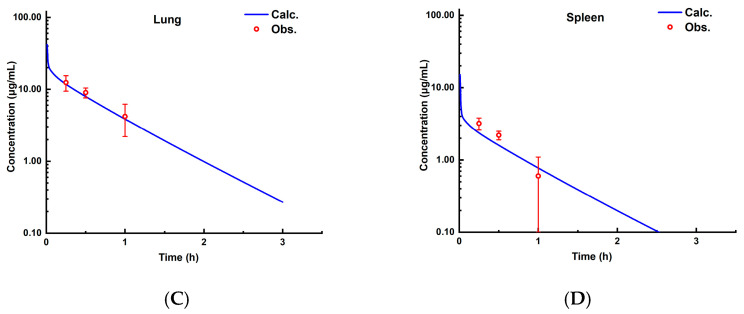
The observed and predicted concentrations of aztreonam in the kidney (**A**), liver (**B**), lung (**C**), and spleen (**D**) in rats (i.v.) of 20 mg/kg, (mean ± SD, *n* = 6).

**Figure 6:**
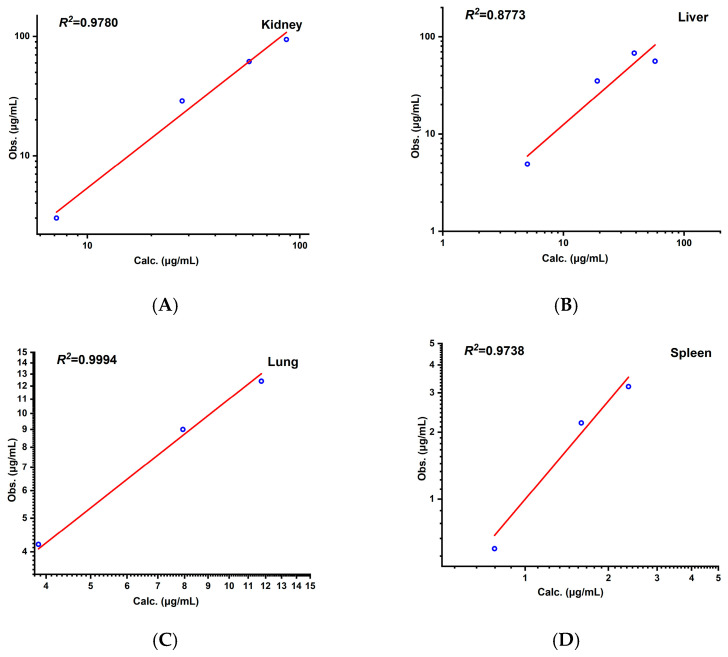
Correlation between observed and predicted values of aztreonam in kidney (**A**), liver (**B**), lung (**C**), and spleen (**D**) in rats (i.v.) of 20 mg/kg.

The experimentally measured *K_p_* values for the kidney, liver, lung, and spleen at four time points (0.25 h, 0.5 h, 1 h, and 2 h) in the rats were calculated based on literature-reported tissue concentration data and compared with the model predictions for calibration. The results demonstrated that all of the FEs for tissue concentrations at each time point (Figure 5) fell within 0.3–3 (see Figure 4). The AFE for the kidney, liver, lung, and spleen were 1.19, 0.75, 0.91, and 0.99, respectively, with an AAFE < 1.5 and *R*^2^ > 0.85 (Figure 6), indicating high reliability of the model in predicting the tissue distribution.

The measured *K_p_* values of the rat kidneys, livers, lungs, and spleens (2.6, 2.3, 0.038, and 0.08, respectively) were obtained from the literature database. Compared with the model-predicted values (3.0, 2.5, 0.4, and 0.08 for kidneys, livers, lungs, and spleens, respectively), the relative errors were all less than 15% (14.5%, 10.3%, 4.7%, and 3.0% for each organ, respectively), thereby verifying the reliability of the calculation method (Table 5). The results demonstrated that the pronounced tissue distribution of aztreonam in the kidneys (*K_p,ra_*_t_ = 2.8, *K_p,human_* = 2.0) was consistent with its pharmacokinetic behavior, in which aztreonam is primarily excreted through glomerular filtration. Additionally, the high renal exposure might be linked to its antibacterial targets (e.g., urinary tract infections). 

For the tissues that have not been directly verified, the *K_p_* values of the remaining tissues (e.g., brain, heart, muscle) were uniformly calculated using the Rodgers and Rowland method embedded in the software. The core input parameters included aztreonam’s physicochemical properties (*Log P*, *pKa*) and species-specific tissue composition data. The results revealed that the *K_p_* values of aztreonam in reproductive system-related tissues were second only to those in the liver and kidneys, which aligns with its clinical characteristics reported in the literature [28,29]. For instance, during the treatment of bacterial prostatitis, the prostate tissue concentration of aztreonam can reach 2.1 times that of plasma [28]. Additionally, in gynecological surgeries, the drug exposure in ovarian tissues was significantly higher compared to cephalosporins (e.g., cefmenoxime) [29]. The relatively high *K_p_* value in lung tissues (second only to reproductive tissues) further corroborates the efficacy advantage of aztreonam in treating pulmonary infections [30].

### 3.3. Cross-Species Extrapolation Verification

**Figure 7:**
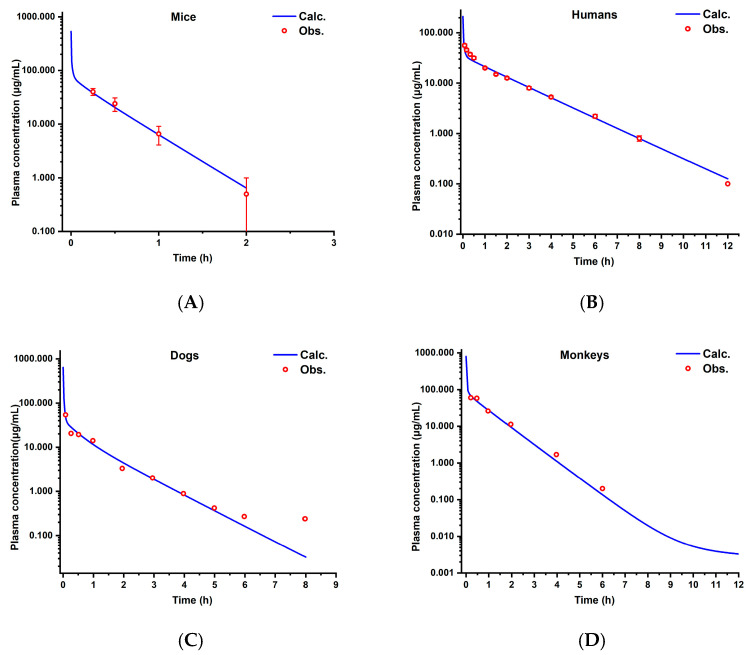
The observed and predicted concentrations of aztreonam in mice (**A**), humans (**B**), dogs (**C**), and monkeys (**D**).

**Figure 8:**
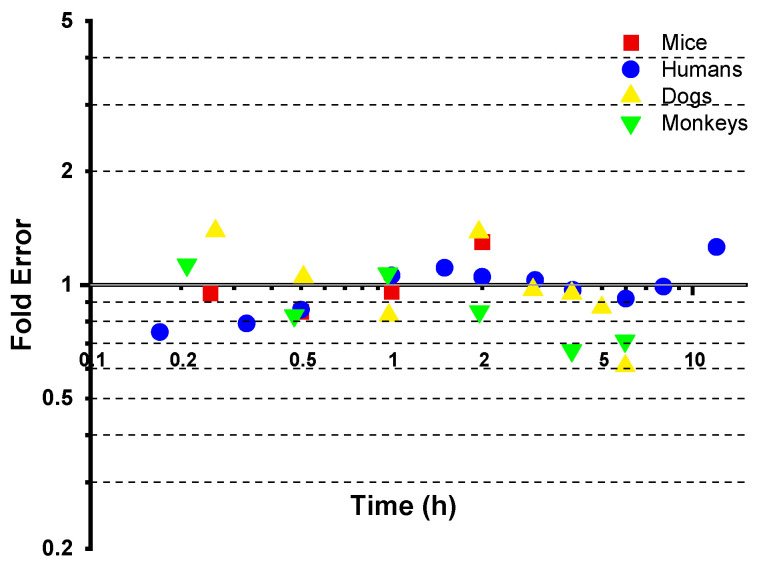
The fold error of all of the observed and predicted concentration points in the mouse, human, dog, and monkey models.

**Figure 9:**
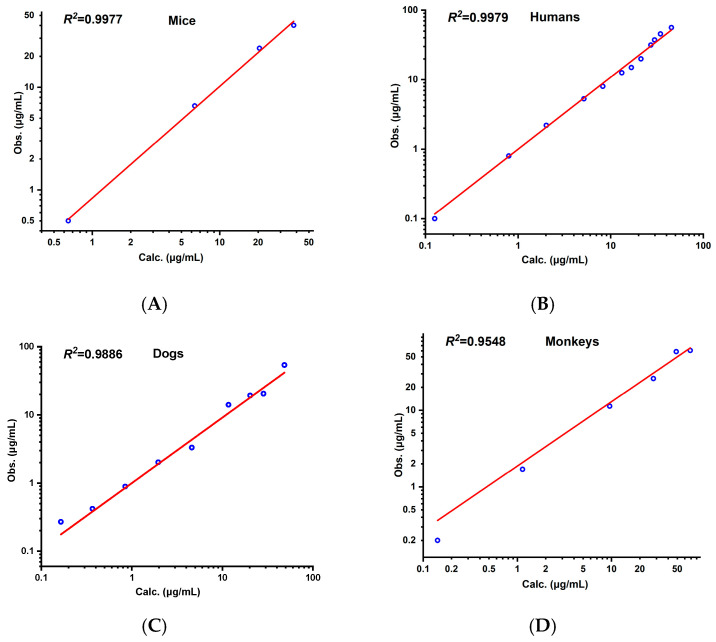
Correlation between observed and predicted values of aztreonam in mice (**A**), humans (**B**), dogs (**C**), and monkeys (**D**).

After the establishment of the PBPK model for rats, the models for mice, humans, monkeys, and dogs were successively built. As shown in Figure 7, the prediction curves and observed values for the mice, humans, monkeys, and dogs are presented in sequence. The FE at each time point is shown in Figure 8, and all were within the range of 0.3–3. The *R*^2^ values of the fitting curves of the predicted values and observed values for the four species were successively 0.98, 0.95, 0.93, and 0.98 (see Figure 9), all of which were >0.80. The AFE values were 1.00, 0.96, 0.97, and 0.86, respectively, and the AAFE values were 1.14, 1.13, 1.21, and 1.24, respectively, all of which were <2.0.

In the mice, the model’s prediction of the AUC showed high reliability (Table 6). The ARE of the *AUC*_0–_*_t_* and *AUC*_0–*∞*_ were both approximately 4%, and the prediction error of the *CL* was only 3.2%, indicating that the model could better capture the drug exposure and clearance mechanisms of mice. However, the predicted value of the *V_ss_* in mice was significantly lower than the measured value (ARE = 27.5%), which might be due to the large standard deviation (SD) of the last sampling point in the literature, resulting in an inaccurate calculation of the *AUC*_0–*∞*_ for the terminal phase extrapolation and thereby affecting the estimation of the *V_ss_*. The PBPK model based on humans performed more robustly. The prediction error of the *AUC* was controlled within 5%, the predicted value of the *CL* differed by 6.4% from the measured value, and the ARE of the *V_ss_* was only 0.04 compared with the measured value.

In dogs (25 mg/kg dose), the ARE between the predicted and observed *V_ss_* was 28.1%, potentially due to uncertainty in the terminal phase data and missing key parameters. The large ARE at the last sampling time point (e.g., 48 h) may introduce errors in extrapolating the *AUC* and mean residence time (MRT), amplifying the *V_ss_* prediction bias. Regarding the unmeasured plasma protein binding in dogs (assumed *f_up_* = 0.33, as with rats), if the actual *f_up_* value is lower, the *K_p_* may be underestimated. Despite the ~30% error in the *V_ss_*, the prediction errors for the *AUC* and *CL* were <16% (Table 7), with an acceptable FE for the plasma concentration points, AFE, and AAFE, indicating that the model’s overall reliability in dogs meets the early development criteria.

In the monkeys (20 mg/kg dose), the predicted *V_ss_* was underestimated by 23.5% compared to the measured value, which might be mainly attributed to the limitations of the terminal phase data and the absence of parameters. However, the prediction errors of the *AUC* and *CL* for the monkeys were both less than 10% (Table 7), verifying the core predictive ability of the model for exposure and clearance rates. Overall, the deviation of the *V_ss_* between dogs and monkeys was mainly caused by the extrapolation error of the terminal phase data and the absence of species-specific parameters (*f_up_*, *R_bp_*), but all errors were controlled within 30%, supporting the rationality of the model under the current data conditions.

### 3.4. Results of Parameter Sensitivity Analysis

**Figure 10:**
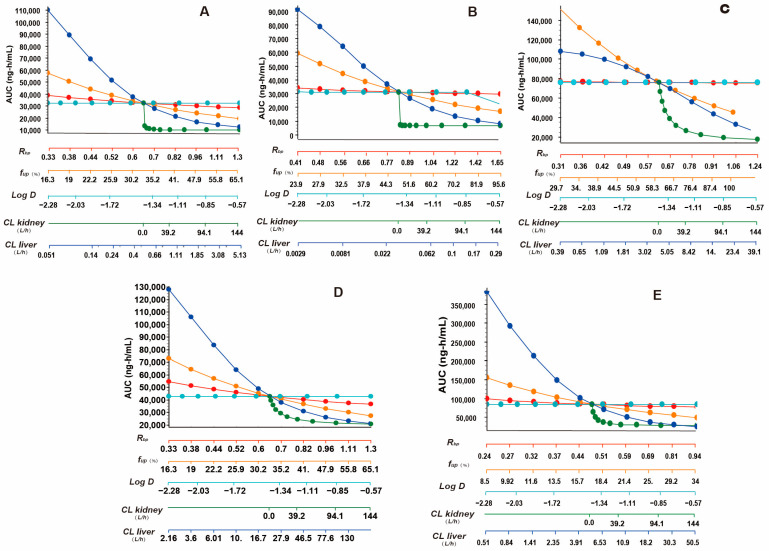
Sensitivity of predicted *AUC*_0–*∞*_ of aztreonam to changes in *R_bp_*, *f_up_*, *Log D*, *CL_liver_*, and *CL_kidney_* of PBPK model in rat (**A**), mouse (**B**), human (**C**), dog (**D**), and monkey (**E**).

To clarify the key parameters of the pharmacokinetic characteristics of aztreonam, a sensitivity analysis was conducted on the model parameters (including *R_bp_, f_up_*, *Log D*, *CL_liver_* and *CL_kidney_*) of rat, mouse, human, dog, and monkey models, with a focus on their contribution to the *AUC*_0–*∞*_ (Figure 10).

Among them, the highest sensitivity of the *CL_kidney_* is consistent with its role as the main clearance pathway of aztreonam. The sensitivity of the *f_up_* shows species dependence. In the human model, the slope of the *f_up_* exceeds that of the *CL_liver_*, while in rodents (rats and mice), the sensitivity of the *f_up_* is lower than that of the *CL_liver_*. The *R_bp_* has a moderate impact on the *AUC*_0–*∞*_ in dogs, which is speculated to be related to the potential binding ability of dog red blood cells to the drug, while in the human model, the sensitivity of the *R_bp_* can be ignored. The *Log D* has the lowest sensitivity in all species, indicating that the tissue distribution of aztreonam is less dependent on its lipophilicity. Except for *Log D*, the other parameters (*R_bp_*, *f_up_*, *CL_liver_*, *CL_kidney_*) show varying degrees of changes in the fitting of the *AUC*_0–*∞*_ in rats, mice, and monkeys, suggesting that the model’s dependence on key physiological parameters is reasonable. The renal and hepatic clearance rates are the core regulatory factors of aztreonam exposure, and the differences in the sensitivity of the *f_up_* and *R_bp_* among species reflect the species heterogeneity of the plasma protein binding and red blood cell distribution. This result provides priority guidance for cross-species model calibration.

## 4. Discussion

Aztreonam, the sole monocyclic β-lactam antibiotic in clinical use, has recently regained significant attention due to its synergistic combination with β-lactamase inhibitors.This study constructed a PBPK model for aztreonam to systematically reveal its pharmacokinetic characteristics and tissue distribution patterns. Moreover, it explored the sensitivity of key parameters to the model predictions. We discuss the following four aspects separately: the model validation, parameter sensitivity, species differences, and the limitations of the study.

### 4.1. Model Validation and Cross-Species Applicability

The model successfully integrated experimental data from rats, mice, dogs, monkeys, and humans. Especially in humans, rats, and mice, the model’s prediction errors for the *CL* and *AUC*_0–*∞*_ were less than 15%, which was significantly better than that of the traditional compartmental models (typical errors > 30%) [27].

This result validates the advantages of the PBPK model in antibiotic PK studies by integrating physiological parameters and mechanistic clearance pathways (such as renal excretion), allowing it to achieve high-precision extrapolation from preclinical to clinical stages. Moreover, the high distribution feature of aztreonam in the kidneys (*K_p_* = 2.0–3.0) is highly consistent with its high efficacy in treating urinary tract infections clinically [31]. The predicted renal tissue exposure by the model is 2–3 times that of plasma, which is directly related to the clearance mechanism of the drug via renal excretion, providing a quantitative basis for optimizing dosing regimens.

### 4.2. Insights from Parameter Sensitivity Analysis

The sensitivity analysis indicates that the renal *CL_kidney_* and *CL_liver_* are the core parameters regulating the exposure amount (*AUC*_0__–*∞*_) of aztreonam, while the contribution of the octanol–water partition coefficient (*Log D*) is extremely low. This phenomenon may be due to the fact that aztreonam enters the systemic circulation through an intravenous injection, and its initial distribution is dominated by the blood flow rate and active transport rather than passive diffusion [32] (*Log D* reflects the lipid solubility diffusion ability). The *Log D* of aztreonam predicted by ADME Predictor (about −1.14 at pH 7.4) indicates that its tissue penetration depends on the cellular bypass pathway and transporter-mediated process, which is consistent with the pharmacokinetic behavior of similar intravenous hydrophilic drugs (such as ceftriaxone and vancomycin). Moreover, the results of the sensitivity parameter analysis show that the sensitivity of the *f_up_* varies among species, and the sensitivity of the *f_up_* in the human model is higher than that in rodents, suggesting that the *R_bp_* may be a potential source of error for extrapolation across species.

### 4.3. Dose-Dependent Pharmacokinetics and Species-Specific Linearity

While the PBPK model assumes linear pharmacokinetics for aztreonam across species, the observed differences in the AUC_0_→∞/dose ratios between 20 mg/kg and 50 mg/kg in rats warrant discussion. The slight deviation in the dose proportionality may stem from experimental variability, particularly in terminal phase sampling, which affects extrapolated AUC values. For instance, the higher variability in the 50 mg/kg group (SD = 5.37 vs. 5.35 for 20 mg/kg) and the exclusion of an outlier at 2 h likely contributed to the apparent nonlinearity. Importantly, the model’s predictions for both doses remained within acceptable error thresholds (ARE < 30%), supporting its robustness. In contrast, human clinical data consistently demonstrate linear pharmacokinetics due to broader sampling windows and lower inter-individual variability. This discrepancy highlights the challenges of extrapolating preclinical rodent data to humans and underscores the need for rigorous terminal phase sampling in animal studies. Future work could incorporate population PK approaches to better characterize the inter-dose variability in rodents and refine the model assumptions.

### 4.4. Interspecies Variability and Model Limitations

Despite overall compliance with validation criteria, discrepancies in the *V_ss_* predictions persisted for dogs and monkeys (ARE = 23.5–28.1%). These deviations may stem from species-specific factors, such as dogs’ higher muscle mass proportion (40–45% vs. 35–40% in humans) and the potential underestimation of the *V_ss_* due to hydrophilic drug retention in muscle tissue [20]. In monkeys, the incomplete representation of hepatobiliary transporter activity in the model may contribute to prediction gaps [33]. Future refinements should incorporate species-specific parameters (e.g., *f_up_*, primate tissue distribution data). Furthermore, the variability in terminal elimination-phase data highlights the need for extended sampling or population pharmacokinetic approaches to enhance the parameter estimation accuracy.

### 4.5. The Limitations of This Study

Although this study successfully constructed a cross-species PBPK model for aztreonam and revealed its pharmacokinetic characteristics, several limitations remain. Firstly, the physiological parameters of some species (such as dogs and monkeys) relied on the default values from the literature, which may introduce species-specific biases. Secondly, the model lacks distribution data for key target tissues (such as cerebrospinal fluid and prostate), limiting its application in complex infection scenarios. Additionally, the potential impact of β-lactamase inhibitors on tissue penetration was not investigated, making it difficult to assess the pharmacokinetic interactions in combination therapy. To address these issues, future research could focus on the following three aspects: firstly, expanding to a multi-drug dynamic model to quantify the impact of β-lactamase inhibitors on aztreonam tissue exposure, providing an optimized strategy for clinical combination therapy; next, incorporating a pharmacokinetic analysis of infection-related deep tissues (such as bone and peritoneal fluid) to enhance the model’s predictive ability for severe infections like sepsis or osteomyelitis; finally, combining in vitro–in vivo extrapolation (IVIVE) techniques and in vitro transporter inhibition experiments to precisely quantify the clearance process mediated by active transport, reducing the reliance on empirical parameters. These improvements will further enhance the biological mechanism basis and clinical translational value of the model.

## 5. Conclusions

The multi-species PBPK model of aztreonam established in this study not only clarified its renal-targeted distribution characteristics and clearance mechanism but also revealed the potential driving factors of inter-species pharmacokinetic differences (such as *CL_kidney_*, *f_up_*). This work could support the development of new antibiotics similar to aztreonam and improve combination therapies.

## Figures and Tables

**Table 1:** Summary of the pharmacokinetic data of aztreonam in mice, dogs, and monkeys.

Reference	Subject	Dosing Regimen
Kita et al., 1986, [12]	SD rats weighing 210 to 250 g, male (*n* = 6)	Intramuscularly to rats (10 mg/mL, 0.2 mL/100 g)
	ICR mice weighing 20 to 25 g, 7-week-old male Jcl (*n* = 6)	A single dose of 20 mg of the aztreonam per kg of body weight was administered subcutaneously to mice (2 mg/mL, 0.1 mL/10 g)
Swabb et al., 1983, [14]	Healthy male subjects, a mean age of 28 years (range 21 to 30), mean height of 177 cm (range 168 to 187), and mean wight of 73.3 kg (range 69.2 to 90.1) (*n* = 4)	500 mg doses of aztreonam administered as single 2 min intravenous infusions
Kripalani et al., 1984, [13]	Young adult male purebred beagles (9 to 11 kg) (*n* = 4)	Single 25 mg/kg doses of aztreonam i.v.
Kita et al., 1986, [12]	Female cynomolgus monkeys weighing 2.8 to 3.7 kg (*n* = 3)	20 mg/mL per kg

**Table 2:** Summary of input parameters for aztreonam PBPK model.

Compound Parameter	Aztreonam
	Predicted Value
Molecular weight	435.43
Oil–water partition coefficient (*log P*)	−1.141
Water solubility (mg/mL) (pH 7.4)	1.187
Plasma free fraction (*f_up_*) (%)	59.4 (human) 32.568 (rat) 47.787 (mouse)
Dissociation constant (*pKa*)	4.09; −0.56; −5.27
Blood–plasma concentration ratio (*R_b__p_*)	0.997 (human) 0.65 (rat) 0.827 (mouse)

**Table 3:** Physiological parameters of PBPK models in rats, mice, humans, dogs, and monkeys.

Tissue	Rats (0.25 kg)		Mice (0.025 kg)		Humans (70 kg)		Dogs (10 kg)		Monkeys (4 kg)	
	Volume (mL)	Blood Flow (mL/s)	Volume (mL)	Blood Flow (mL/s)	Volume (mL)	Blood Flow (mL/s)	Volume (mL)	Blood Flow (mL/s)	Volume (mL)	Blood Flow (mL/s)
Lung	2.1	0.7990	0.1583	0.1135	914.4144	85.7230	86.6667	18.4029	34	8.6929
Arterial Supply	5.6	0.7990	0.57	0.1135	189.8027	85.7230	300	18.4029	107	8.6929
Venous Return	11.3	0.7990	1.13	0.1135	3619.6054	85.7230	600	18.4029	213	8.6929
Adipose	10	0.0067	1.9105	0.0013	23,762.7391	8.3374	1637.5546	0.5830	437	0.2670
Muscle	122	0.1251	9.2219	0.0152	17,027.0270	8.9701	4385.2065	4.1659	2000	1.5
Liver	10.3	0.1967	1.6636	0.0335	13,440.0901	21.6172	303.2710	5.1501	93.46	2.4225
ACAT Gut	0	0.1250	0	0.0250	0	12.0432	0	3.5965	0	1.6667
Spleen	0.6	0.01	0.1008	0.0015	142.1316	2.4960	24.6679	0.4167	2.85	0.0476
Heart	1.2	0.0650	0.1092	0.0047	265.2499	3.4004	76.6990	0.9	13.6	0.7572
Brain	1.2371	0.0217	0.4165	0.0076	1411.5727	12.6419	76.2910	0.8643	89	1.2291
Kidney	3.7	0.1533	0.3893	0.0213	231.8303	14.9815	50	3.6	12.4	1.1978
Skin	40.0	0.0957	3.5158	0.0101	1608.1081	3.3887	774.2045	2.3017	400	0.9
Reproductive	2.5	0.0083	0.1480	0.0005	26.3200	0.0971	16.4	0.0574	22	0.077
Red marrow	1.8641	0.0304	0.8320	0.0136	965.3183	5.0855	135	0.3933	36	0.18
Yellow marrow	4.1480	0.0068	0.5245	0.0009	2683.2505	1.4136	64.6	0.0188	102	0.051
Rest of body	24.421	0.0884	1.3735	0.0050	10,989.8249	5.7896	736.7420	0.3684	222.6144	0.1113

**Table 4:** Observed and predicted pharmacokinetic parameters in rats (i.v.) of 50 mg/kg (A) and 20 mg/kg (B).

Parameters	Rats 50 mg/kg				Rats 20 mg/kg			
	Observed	Predicted	RPE (%)	ARE (%)	Observed	Predicted	RPE (%)	ARE (%)
*AUC*_0–*t*_ (μg·h/mL)	53.61	55.40	3.4	3.4	37.69	31.83	−15.5	15.5
*AUC*_0–*∞*_ (μg·h/mL)	53.62	55.43	3.3	3.3	37.93	32.36	−14.7	14.7
*CL* (L/H)	0.21	0.216	2.4	2.4	0.122	0.145	18.9	18.9
*V_ss_* (L)	0.06	0.068	6.3	6.3	0.074	0.09	21.6	21.6

**Table 5:** Summary of aztreonam *K_p_* values.

Tissue	Predicted *K_p_* Values by Observed *K_p_* Values in Lukacova (Rodgers-Single) Method Using GastroPlus™	Observed *K_p_* Values in Rats	Predicted *K_p_* Values by Observed *K_p_* Values in Humans
Lung	0.40	0.38	0.32
Adipose	0.09	/	0.07
Muscle	0.29	/	0.24
Liver	2.5	2.3	2.03
Spleen	0.08	0.08	0.06
Heart	0.36	/	0.29
Brain	0.30	/	0.24
Kidney	3.0	2.6	2.43
Skin	0.28	/	0.23
Reproductive organ	0.44	/	0.36
Red marrow	0.25	/	0.20
Yellow marrow	0.09	/	0.07
Rest of body	0.34	/	0.28

**Table 6:** Observed and predicted pharmacokinetic parameters in mice and humans.

Parameters	Mice 20 mg/kg				Humans 500 mg			
	Observed	Predicted	RPE (%)	ARE (%)	Observed	Predicted	RPE (%)	ARE (%)
*AUC*_0–_*_t_* (μg·h/mL)	30.05	31.26	4.0	4.0	79.78	75.76	−5.0	5.0
*AUC*_0–*∞*_ (μg·h/mL)	30.24	31.54	4.3	4.3	79.97	76.04	−4.9	4.9
*CL* (L/H)	0.017	0.016	−3.2	3.2	6.16	6.56	6.4	6.4
*V_ss_* (L)	0.007	0.005	−27.5	27.5	13.190	13.140	−0.4	0.4

**Table 7:** Observed and predicted PK parameters in dogs and monkeys.

Parameters	Dogs 25 mg/kg				Monkeys 20 mg/kg			
	Observed	Predicted	RPE (%)	ARE (%)	Observed	Predicted	RPE (%)	ARE (%)
*AUC*_0–*t*_ (μg·h/mL)	37.10	42.96	15.8	15.8	77.68	84.831	9.2	9.2
*AUC*_0*-∞*_ (μg·h/mL)	37.47	43.00	14.8	14.8	77.88	84.821	8.9	8.9
*CL* (L/H)	6.338	5.570	−12.1	12.1	0.845	0.775	−8.3	8.3
*V_ss_* (L)	6.480	4.660	−28.1	28.1	0.835	0.639	−23.5	23.5

## Data Availability

All data are presented in the article and Supplementary Materials.

## Supplementary Materials

The following supporting information can be downloaded at https://www.mdpi.com/article/10.3390/pharmaceutics17060748/s1.
