# Supplementary material for: Tissue Distribution and Pharmacokinetic Characteristics of Aztreonam Based on Multi-Species PBPK Model
Source: Pharmaceutics. 2025 Jun 6;17(6):748. doi: 10.3390/pharmaceutics17060748 (PMC12196478; doi:10.3390/pharmaceutics17060748)
Supplement: Supplementary file 1 [file pharmaceutics-17-00748-s001.zip › pharmaceutics-3567991-supplementary.pdf]

Supplementary Materials

List of Supplementary Figures

Figure S1. Representative Chromatograms of Aztreonam and IS in Rat Plasma (LLOQ, 5 ng/mL).

Figure S2. Mass Spectra of Aztreonam （*m/z* 434.1 → *m/z* 95.9） and IS（*m/z* 462.0 → *m/z* 151.9）

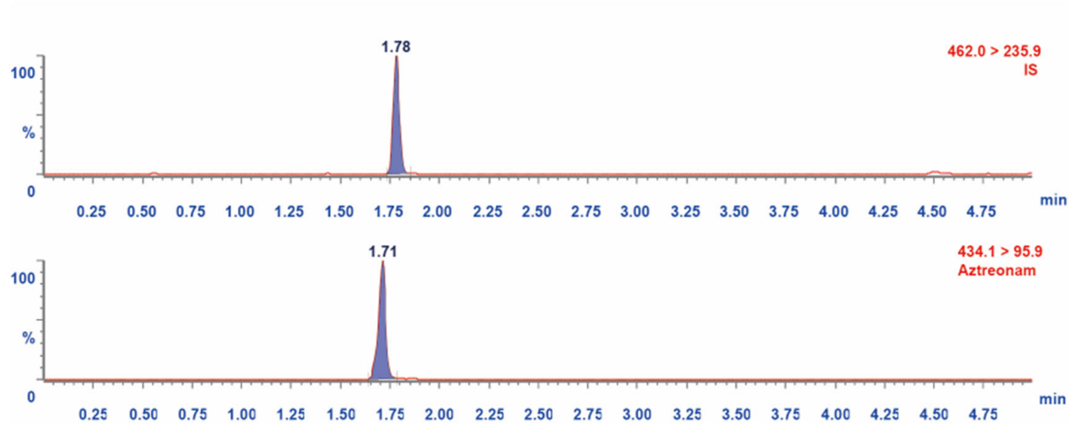

Figure S1. Representative Chromatograms of Aztreonam and IS in Rat Plasma (LLOQ, 5 ng/mL).

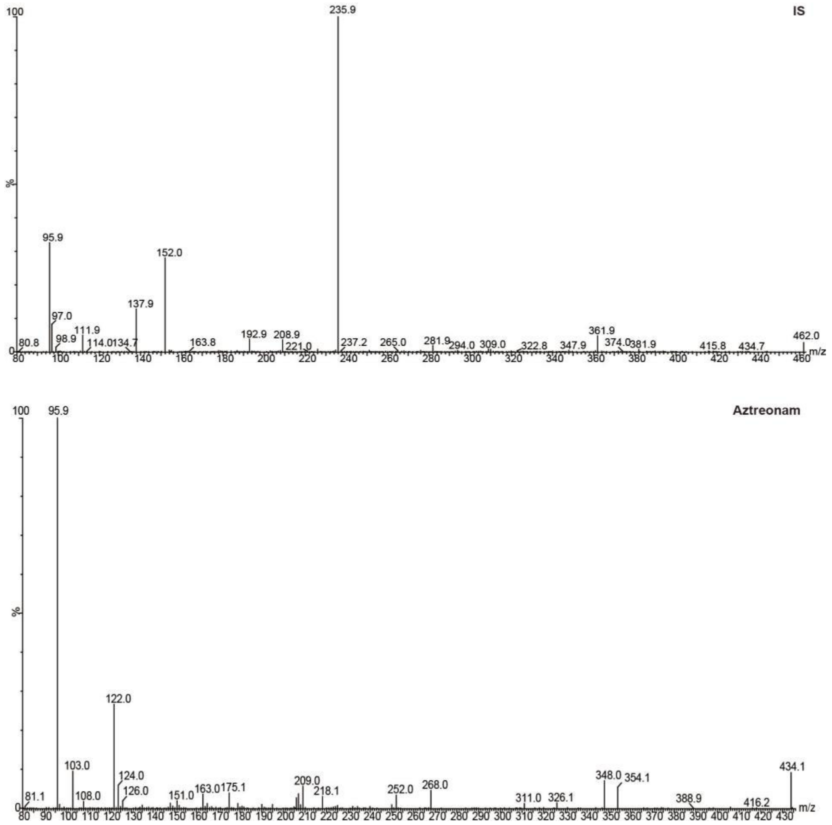

Figure S2. Mass Spectra of Aztreonam （*m/z* 434.1 → *m/z* 95.9）and IS（*m/z* 462.0 → *m/z* 151.9）

Validation of the method for the analysis of plasma aztreonam in SD rats

Bioanalytical Method Validation

The established bioanalytical method was validated in accordance with the M10: Bioanalytical Method Validation and Study Sample Analysis guidelines issued by the International Council for Harmonization of Technical Requirements for Pharmaceuticals for Human Use (ICH).

Selectivity

Blank plasma samples (50 μL) from six different individuals were deproteinized with 200 μL of methanol-acetonitrile (9:1, v/v) and analyzed to evaluate potential interference from endogenous components at the retention times of aztreonam and the internal standard (IS). The peak area of interfering

substances at the aztreonam retention time in blank samples was required to be less than 20% of the peak area of aztreonam in the lower limit of quantification (LLOQ) samples. Similarly, interference at the IS retention time was required to be less than 5% of the IS peak area in LLOQ samples.

Calibration Curve and Quantification Limit

A series of standard working solutions (50, 100, 500, 1500, 3000, 5000, 7000, and 10,000 ng/mL) were prepared. Calibration standards were processed according to ***Section 2.3.2 Sample Preparation*** and analyzed to generate chromatograms and peak areas. A linear regression model with $1/x^2$ weighting was applied to the ratio of aztreonam to IS peak areas (Y-axis) versus nominal concentrations (X-axis). The calibration curve demonstrated linearity within the range of 5–1000 ng/mL, with an LLOQ of 5 ng/mL and an upper limit of quantification (ULOQ) of 1000 ng/mL.

Precision and Accuracy

Intra-day precision and accuracy: Five replicates of LLOQ (5 ng/mL), low (15 ng/mL), medium (180 ng/mL), and high (800 ng/mL) quality control (QC) samples were prepared and analyzed on the same day. The percentage deviation (% Dev) and relative standard deviation (RSD) were calculated. For LLOQ samples, accuracy (% Dev) was required to be within ±20%, and precision (RSD) ≤20%. For other QC levels, accuracy and precision were required to be within ±15% and ≤15%, respectively.

Inter-day precision and accuracy: Three batches of QC samples were prepared and analyzed over at least two days. Inter-day RSD values were calculated, with acceptance criteria identical to those for intra-day evaluations.

Matrix Effect

Six batches of blank plasma from different individuals were used to prepare QC samples at 15 ng/mL and 800 ng/mL (n=3 per batch). Samples were analyzed using a concurrently prepared calibration curve to assess the impact of endogenous matrix components. The accuracy (% Dev) and precision (RSD) of

matrix effect samples were required to be within ±15% and ≤15%, respectively.

Dilution Integrity

Samples exceeding the ULOQ (1000 ng/mL) were diluted 2-fold and 100,000-fold with blank plasma to obtain concentrations of 800 ng/mL and 10 ng/mL (*n*=5). Analyte concentrations were determined using a concurrent calibration curve. Accuracy (%Dev) and precision (RSD) for diluted samples were required to be within ±15% and ≤15%, respectively.

Stability

Short-term stability: QC samples (800 ng/mL and 15 ng/mL) were stored at room temperature under light-protected conditions for 4 hours prior to processing. Accuracy (% Dev) of stability samples was required to be within ±15%.

Long-term stability: QC samples (800 ng/mL and 15 ng/mL) were stored at −70 °C for 70 days, thawed, and analyzed. Accuracy (% Dev) was required to be within ±15%.

Freeze-thaw stability: QC samples underwent three freeze-thaw cycles (−70 °C, ≥12 hours per cycle). Accuracy (% Dev) was required to be within ±15%.

Post-processing stability: Processed samples stored in the autosampler at 10 °C for 28 hours were reanalyzed, with accuracy (% Dev) required to be within ±15%.

Injection Reproducibility

All precision and accuracy batches meeting acceptance criteria were re-injected. The accuracy of LLOQ samples was required to be within ±20%, while other QC samples were required to be within ±15%, confirming that samples remain stable and suitable for re-injection following instrument interruptions.

Results

Selectivity

In blank plasma samples from six different Sprague-Dawley (SD) rats, the peak areas of interfering substances at the retention time of aztreonam were less than 20% of the corresponding peak area in the lower limit of quantification (LLOQ) samples. Similarly, interference peaks at the retention time of the internal standard (IS) were less than 5% of the IS peak area in LLOQ samples. These results confirm that endogenous components in rat plasma do not interfere with the accurate quantification of aztreonam, demonstrating high selectivity of the analytical method.

Calibration Curve and Quantification Limit

Calibration standards for aztreonam in SD rat plasma were prepared at concentrations of 2, 5, 10, 50, 150, 300, 500, 700, and 1000 ng/mL, with the 2 ng/mL concentration serving as an anchor point for linear regression (excluded from quantitative analysis). Validation results demonstrated excellent linearity (r^2 > 0.999) over the quantification range of 5–1000 ng/mL, indicating the method's reliability for accurately quantifying aztreonam in biological samples within this range. The measured concentrations of calibration standards, along with the linear regression equations and validation data from three independent analytical batches, are summarized in Tables 3.1 and 3.2.

Table S1 Measured concentration of aztreonam of correction standard samples

Nominal concentration（ng/mL）	Measured concentration（ng/mL）			Average（ng/mL）	Standard deviation(SD)（ng/mL）
	b1	b2	b3		
5	5.081	5.260	5.085	5.142	0.102
10	9.391	9.921	9.696	9.669	0.266
50	48.995	49.464	48.364	48.941	0.552
150	157.757	148.147	155.860	153.921	5.090

300	300.395	287.284	301.774	296.484	7.998
500	501.939	500.032	491.838	497.936	5.367
700	697.033	709.188	723.382	709.868	13.188
1000	994.374	1005.699	978.991	993.021	13.405

*b1, b2, and b3 represent the three analysis batches respectively

Table S2 Linear equation of calibration curve of rat plasma in method verification

Batch	a	b	r^2
b1	0.396644	-0.0457419	0.999804
b2	0.391765	-0.0481825	0.999720
b3	0.427786	-0.00125799	0.999367

Carryover Effect

Carryover of aztreonam and IS was evaluated across three analytical batches by injecting upper limit of quantification (ULOQ) standards followed by blank samples. Results demonstrated that in all three batches, the peak area of aztreonam in blank samples was less than 20% of that in LLOQ samples, while the IS peak area in blanks did not exceed 5% of the IS peak area in LLOQ samples (Table S3). These findings indicate minimal carryover interference from high-concentration samples (e.g., ULOQ) on the accurate quantification of low-concentration samples.

Table S3 Determination results of residues of aztreonam and IS

Sample	IS Area	Aztreonam Area	20% Area of Aztreonam in LLOQ	5% Area of IS in LLOQ
Blank 1	4.41	45.04	181.39	2302.42
Blank 2	10.56	20.52	170.46	2117.57
Blank 3	19.85	48.78	175.05	2013.03

Accuracy and Precision

Three independent precision and accuracy batches were analyzed over three days, each containing calibration standards, LLOQ (5 ng/mL), low QC (LQC, 15

ng/mL), medium QC (MQC, 180 ng/mL), and high QC (HQC, 800 ng/mL) samples (n=5 per level).All results met the predefined acceptance criteria outlined in the above-mentioned requirements. Detailed data are provided in Tables S4 and S5.

Table S4 Accuracy and precision results of intra-lot of QC samples of aztreonam(*n*=5)

Batch	Nominal concentration (ng/mL）	Intra-lot of QC samples（*n*=5）			
		Average（ng/mL）	SD（ng/mL）	RSD（%）	Accuracy（%）
b1	5	5.123	0.262	5.12	102.47
	15	14.549	0.578	3.97	96.99
	180	171.789	3.029	1.76	95.44
	800	750.928	32.399	4.31	93.87
b2	5	4.851	0.223	4.60	97.02
	15	15.376	1.086	7.06	102.51
	180	185.763	5.580	3.00	103.20
	800	811.288	25.571	3.15	101.41
b3	5	4.472	0.105	2.35	89.44
	15	14.406	0.360	2.50	96.04
	180	182.766	6.569	3.59	101.54
	800	791.315	31.272	3.95	98.91

Table S5 Accuracy and precision results of inter-lot of QC samples of aztreonam (*n*=15)

Batch	Nominal concentration（ng/mL）	Inter-lot of QC samples（*n*=15）			
		Average（ng/mL）	SD（ng/mL）	RSD（%）	Accuracy（%）
b1、b2、b3	5	4.815	0.337	7.00	96.31
	15	14.777	0.816	5.52	98.51
	180	180.106	7.906	4.39	100.06
	800	784.510	37.968	4.84	98.06

Matrix effect

Matrix effect samples prepared from six batches of blank plasma (from different SD rat) at concentrations of 800 ng/mL and 15 ng/mL (*n*=3 per batch)

demonstrated accuracies ranging from 92.56% to 97.88%, with precision (RSD) between 0.62% and 5.36%. These results met the acceptance criteria, confirming that endogenous components in rat plasma have minimal and negligible impact on the accuracy of aztreonam quantification. Detailed results are provided in TableS6.

Table S6 Determination results of matrix effect samples of aztreonam (*n*=3)

Nominal concentration（ng/mL）	Individual	Measured concentration（ng/mL）			Average（ng/mL）	SD（ng/mL）	RSD（%）	Accuracy（%）
15	1	14.464	13.783	14.351	14.199	0.365	2.57	94.66
	2	15.148	14.143	14.755	14.682	0.506	3.45	97.88
	3	15.022	13.633	14.398	14.351	0.696	3.77	95.68
	4	14.712	14.714	14.440	14.622	0.158	1.08	92.56
	5	13.938	15.308	14.006	14.417	0.772	5.36	96.11
	6	14.458	14.673	14.405	14.512	0.142	1.31	96.75
800	1	748.229	742.073	737.863	742.722	5.213	0.70	92.84
	2	753.836	750.775	757.408	754.006	3.320	0.62	94.25
	3	776.020	733.858	778.208	762.695	24.998	4.10	95.34
	4	779.691	700.485	741.397	740.524	39.610	5.35	97.48
	5	772.609	732.256	721.319	742.061	27.014	1.04	92.76
	6	737.832	773.322	745.906	752.353	18.603	2.58	94.05

Dilution Integrity

Samples diluted 2-fold and 100,000-fold with blank pooled SD rat plasma showed mean accuracies of 88.84% (precision: 1.49%) and 88.95% (precision: 3.08%), respectively. These outcomes complied with the requirements outlined in *Section **Dilution Integrity***, demonstrating that diluting rat plasma samples 10- to 100,000-fold with blank pooled plasma during pharmacokinetic (PK) sample analysis has negligible impact on the reliability of aztreonam quantification.

Stability

Long-Term Stability

Aztreonam plasma stability samples stored at -70 °C for 25, 52, and 70 days

were analyzed. Results showed that the mean accuracies of LQC (*n*=3) were 97.48%, 94.80%, and 103.45%. The mean accuracies of HQC (*n*=3) were 96.38%, 93.52%, and 99.50%.All values met the acceptance criteria, confirming the stability of aztreonam in rat plasma for up to 70 days at -70 °C.

Freeze-Thaw Stability

After three freeze-thaw cycles of plasma samples stored at -70 °C, the mean accuracies of aztreonam were 94.80% (15 ng/mL) and 97.63% (800 ng/mL) (*n*=3). These results met the acceptance criteria, demonstrating stability under repeated freeze-thaw conditions.

Short-Term Stability at Room Temperature

Aztreonam plasma samples (15 ng/mL and 800 ng/mL, *n*=3) stored at room temperature under light protection for 4 hours exhibited mean accuracies of 99.32% and 102.64%, respectively. These outcomes satisfied the requirements, proving short-term stability.

Autosampler Stability

QC samples injected on the previous day were reanalyzed using freshly prepared calibration standards and QC samples. The mean accuracy of all QC levels remained within ±15%, even after a 27-hour post-preparation storage period in the autosampler. This confirms the stability of processed samples under autosampler conditions.

Reinjection Reliability

Reanalysis of all validated precision and accuracy batch samples showed that the accuracy deviations of LLOQ samples within ±20%.The accuracy deviations of Other QC samples (LQC, MQC, HQC) within ±15%.These findings demonstrate that samples can be reliably re-injected within the validated stability window, even in cases of instrument interruption.

Conclusion

The LC-MS/MS-based bioanalytical method for quantifying aztreonam in Sprague-Dawley rat plasma was comprehensively validated following ICH M10 guidelines, demonstrating robustness and reliability for preclinical pharmacokinetic studies. The method exhibited high selectivity, with endogenous interference at the retention times of aztreonam (*m*/*z* 434.1 → 95.9) and the internal standard (*m*/*z* 462.0 → 151.9) remaining below 20% and 5% of the LLOQ response, respectively. A linear calibration curve ($r^2 > 0.999$) was established over the range of 5–1000 ng/mL, with an LLOQ of 5 ng/mL, ensuring sensitivity for low-concentration measurements. Intra- and inter-day precision (%RSD ≤15%) and accuracy (%Dev ±15%) met predefined criteria across all QC levels, including LLOQ samples. Minimal matrix effects were observed, with accuracies ranging from 92.56% to 97.88% and precision (%RSD) ≤5.36%, confirming consistent performance across plasma batches. Dilution integrity was maintained even at extreme dilutions (2- to 100,000-fold), with accuracies of 88.84%–88.95% and precision (%RSD ≤3.08%). Stability studies further validated the method's practicality, as aztreonam remained stable under short-term (4 h at room temperature), long-term (70 days at −70 °C), freeze-thaw (3 cycles), and autosampler (28 h at 10 °C) conditions. Overall, this method provides a precise, accurate, and reliable tool for characterizing aztreonam pharmacokinetics in preclinical models.
